# Assessment of Active Video Gaming Using Adapted Controllers by Individuals With Physical Disabilities: A Protocol

**DOI:** 10.2196/resprot.7621

**Published:** 2017-06-16

**Authors:** Laurie A Malone, Sangeetha Padalabalanarayanan, Justin McCroskey, Mohanraj Thirumalai

**Affiliations:** ^1^ University of Alabama at Birmingham/Lakeshore Foundation Research Collaborative Birmingham, AL United States

**Keywords:** video games, exercise, physical activity, disability, energy expenditure, enjoyment

## Abstract

**Background:**

Individuals with disabilities are typically more sedentary and less fit compared to their peers without disabilities. Furthermore, engaging in physical activity can be extremely challenging due to physical impairments associated with disability and fewer opportunities to participate. One option for increasing physical activity is playing active video games (AVG), a category of video games that requires much more body movement for successful play than conventional push-button or joystick actions. However, many current AVGs are inaccessible or offer limited play options for individuals who are unable to stand, have balance issues, poor motor control, or cannot use their lower body to perform game activities. Making AVGs accessible to people with disabilities offers an innovative approach to overcoming various barriers to participation in physical activity.

**Objective:**

Our aim was to compare the effect of off-the-shelf and adapted game controllers on quality of game play, enjoyment, and energy expenditure during active video gaming in persons with physical disabilities, specifically those with mobility impairments (ie, unable to stand, balance issues, poor motor control, unable to use lower extremity for gameplay). The gaming controllers to be evaluated include off-the-shelf and adapted versions of the Wii Fit balance board and gaming mat.

**Methods:**

Participants (10-60 years old) came to the laboratory a total of three times. During the first visit, participants completed a functional assessment and became familiar with the equipment and games to be played. For the functional assessment, participants performed 18 functional movement tasks from the International Classification of Functioning, Disability, and Health. They also answered a series of questions from the Patient Reported Outcomes Measurement Information System and Quality of Life in Neurological Conditions measurement tools, to provide a personal perspective regarding their own functional ability. For Visit 2, metabolic data were collected during an initial 20-minute baseline, followed by 40 minutes of game play. The controller (balance board or gaming mat) played was randomly selected. A set of games was played for 10 minutes, followed by 5 minutes of rest, and then another set of games was played for 10 minutes, followed by rest. Quality of game play was observed and documented for each set. During rest, the participant completed questions regarding enjoyment. Following the same procedures, the participant then played the two sets of games using the other version (off-the-shelf or adapted) of the controller. The entire procedure was repeated during Visit 3 with the controller that was not played.

**Results:**

Enrollment began in February 2016 and ended in September 2016. Study results will be reported in late 2017.

**Conclusions:**

We hypothesized that the adapted versions of the Wii Fit balance board and gaming mat would produce greater quality of game play, enjoyment, and energy expenditure in persons with mobility impairments compared to off-the-shelf versions.

**Trial Registration:**

ClinicalTrials.gov NCT02994199; https://clinicaltrials.gov/ct2/show/NCT02994199 (Archived by WebCite at http://www.webcitation.org/6qpPszPJ7)

## Introduction

Physical activity options for individuals with physical disabilities are limited, and existing community fitness programs and available exercise equipment are often inaccessible [1]. With these and other existing barriers (eg, transportation, program cost, staff knowledge), individuals with disabilities are more likely to be physically inactive [2-6] and demonstrate higher rates of obesity [7-11] than their counterparts without disabilities. Development of creative strategies by which to actively engage people with physical disabilities in leisure-time physical activity is vital given the high prevalence of physical inactivity and obesity in this population. One option for increasing the amount of physical activity acquired each week is to replace sedentary behaviors during leisure time with active video game (AVG) play [12-14].

Video game play typically requires only simple pushbutton or joystick actions for player engagement. AVGs, on the other hand, also known as exergames, refer to a category of video games in which game play, progress, and scoring require substantially greater levels of body movement. Since their introduction, AVGs have become popular with people of all ages and have been used in home, community, education, and rehabilitation settings to increase physical activity [13,15-20]. Furthermore, a growing body of literature indicates that AVGs show promise as an enjoyable physical activity alternative for individuals with physical disabilities—one that overcomes some of the common barriers to activity participation such as transportation and facility access [21-26].

It is important to determine whether AVGs can provide a level of physical activity commensurate with achieving health and fitness benefits. A few studies have reported that increases in energy expenditure can be achieved during AVG play in people with physical disabilities, more specifically those with mobility impairments such as cerebral palsy (CP) [27-30], spinal cord injury (SCI) [31,32], and stroke [33-35]. A study by Hurkmans et al [27] measured energy expenditure using indirect calorimetry in adults with CP during Nintendo Wii tennis and boxing games. Participants played in a standing position and achieved moderate intensity exercise during both games. Similarly, two AVG studies on ambulatory youth with CP found that participants were able to achieve moderate intensity exercise with various Nintendo Wii games [28-29] as well as Dance Dance Revolution [28]. A case study on two young adults with CP and one with spina bifida in which participants played Nintendo Wii games and Dance Dance Revolution (adapted for arm use) in a seated position found that only light intensity exercise was achieved during gameplay [30]. Another case study on two young adults with SCI measured heart rate during Nintendo Wii boxing in a seated position and recorded moderate intensity heart rate levels [31]. Additionally, a study of adults with SCI compared seated heavy bag boxing and AVG boxing and found participants achieved moderate intensity exercise levels during both types of boxing and reported AVG boxing to be more enjoyable [32]. Using the Borg Scale of Perceived Exertion, adults post-stroke scored a variety of Nintendo Wii and Sony PlayStation EyeToy AVGs played in a standing position as light to moderate intensity [34]. Two other studies on adults post-stroke using indirect calorimetry during Nintendo Wii [33,35] and Sony Xbox 360 [33] found AVG gameplay while standing to produce moderate intensity exercise. Kafri et al [33] also had participants post-stroke play Nintendo Wii boxing in both a standing and a seated position and found that in the sitting position participants approached anaerobic metabolism.

While it appears that AVGs hold promise as a means for improving health and fitness, there are technical issues associated with their access and utility in individuals with more severe physical disabilities. For instance, the studies noted above for individuals with CP and post-stroke were composed mostly of community ambulators with mild to moderate motor impairments. Unfortunately, however, for individuals with mobility impairments, specifically those who are unable to stand, have balance issues, poor motor control, or cannot use their lower body to perform game activities, gaming hardware for AVG play is typically inaccessible or the game itself offers limited play options [22-23]. AVGs using floorpad game controllers (eg, Dance Dance Revolution, Active Life Outdoor Challenge) have obvious accessibility limitations. The quick and precise motions required for successful AVG play using hand controllers (eg, Sony PlayStation Move motion controller, Nintendo Wii remote and nunchuck) limits their use for many people as well. Systems such as the Microsoft Xbox Kinect, which use a camera-based controller, also pose problems for successful gameplay, as they typically require the player to be standing for the game to function properly.

Rehabilitation engineers and assistive technology specialists have developed a variety of creative and successful adaptations to game controllers and interfaces that allow people with disabilities to play video games. However, successful adaptations of game controllers to allow people with mobility impairments to play AVGs require not only modifications to allow satisfactory game play but also redesigns to assure that the player with a disability experiences a similar level of energy expenditure. Making AVG hardware accessible for people with physical disabilities offers an innovative approach to overcoming a number of barriers to participation and provides an enjoyable and beneficial physical activity option. Our Rehabilitation Engineering Research Center on Interactive Exercise Technologies and Exercise Physiology for Persons with Disabilities (RERC RecTech) team developed two adapted controllers including an adapted balance board for Wii Fit as well as an adapted gaming mat.

Off-the-shelf (OTS) Wii Fit balance boards are designed for play to occur in a standing position. The small platform area (19.5 inches x 12 inches) is only large enough for a player to stand on with feet approximately shoulder width apart. In addition, because the board uses load cells to detect the player’s weight and center of balance, all of the player’s weight must bear on the top of platform. This makes the board less responsive to those who require the use of stabilization devices (eg, cane, walker) that bear on the floor around the platform and not usable for someone seated in a wheelchair. Furthermore, to fully engage in gameplay, the player must lean and maintain balance in all directions (forward, backward, side-to-side). To increase the level of usability for those with various forms of mobility impairment, an adapted balance board was designed to provide a large platform area (40 inches x 38 inches), built-in lateral stabilization supports (ie, handrails), and an adjustable sensitivity for shifting the center of balance.

Like the boards, OTS gaming mats are designed for players who can stand and have little to no lower extremity mobility impairment, having a 3 foot x 3 foot playing surface over which eight controller buttons and two menu buttons are widely distributed. This design poses several issues given that many players with mobility impairments would be better accommodated by playing in a seated position. The large playing surface makes it difficult to reach all of the buttons when seated at a table. Furthermore, the buttons are designed for high actuation force as would be common when used by a standing player, thereby becoming very difficult for use with the hands and/or fingers. In addition, the underlying design of the mat buttons is such that “dead” spots exist in the area of each button, which may not be triggered if a player were to try and depress a button with a couple of fingers rather than a whole foot or hand. To increase the level of usability for players with various forms of mobility impairment, an adapted gaming mat was designed with moveable Velcro buttons that could be positioned more closely to each other, with a gauge for reducing the actuation force required, and elimination of the dead spots resulting in a consistent button response over the entire button area.

Our aim was to compare the effect of off-the-shelf and adapted game controllers on quality of game play, enjoyment, and energy expenditure during active video gaming in persons with physical disabilities, specifically those with mobility impairments (ie, unable to stand, balance issues, poor motor control, unable to use lower extremity for gameplay). The gaming controllers evaluated included off-the-shelf and adapted versions of the Wii Fit balance board and gaming mat.

## Methods

### Design and Setting

All aspects of the study took place at Lakeshore Foundation in Birmingham, Alabama. Lakeshore Foundation is a community organization that provides physical activity, sport, and recreation opportunities for individuals with physical disability and chronic health conditions. Within Lakeshore is the Exercise and Sport Science Laboratory, which houses a variety of equipment dedicated to comprehensive health promotion and sport science research. For the purposes of this study, participants came to the lab a total of three times.

### Participants

Following distribution of a flyer, the project recruitment coordinator answered calls or met with interested individuals. At that time, she reviewed the inclusion and exclusion criteria with them using a screening form to determine if they were eligible to participate. Our aim was to enroll 80 participants (15 youth, aged 10-17 years; 65 adults, aged 18-60 years) into the study. Participants were included in the study if they had a confirmed diagnosis of lower extremity mobility limitation (eg, spina bifida, CP, muscular dystrophy, 1 year post-SCI, multiple sclerosis, stroke, or limb loss) with partial or full use of upper extremities and use of an assistive device (eg, cane, walker, wheelchair) or problems with gait, balance, and/or coordination. Participants were excluded if they had an unstable cardiovascular condition, a visual impairment that interferes with playing video games (eg, complete blindness; inability to read game commands on a 52-inch television screen from a distance of 10 feet), or weighed over 350 lbs including their assistive device.

### Procedures

#### Visit 1

During the first visit, informed consent/assent was obtained and demographic and health history information was documented. We conducted an assessment of each participant’s functional ability as described below. In addition, participants were familiarized with the equipment (Cosmed K4b2 portable metabolic system) used for the study and the video games that would be played during subsequent visits. Participants played a portion of or the entire game for all those that would be used during testing.

For assessment of physical function, which was conducted during the first visit, each participant performed 18 functional tasks from the International Classification of Functioning, Disability and Health (ICF) [36,37]. Participants completed each task individually and were scored according to their difficulty in completing the task on a scale ranging from 0-4. As defined in the ICF manual, the scoring was as follows: 0=No difficulty, 1=Mild difficulty, 2=Moderate difficulty, 3=Severe difficulty, and 4=Complete difficulty. The specific ICF tasks selected for use in this study were based on a consensus among the research staff. Following observations during pilot testing, staff selected mobility activities listed in the ICF that had the potential to be required for AVG play (eg, standing, reaching, throwing, and jumping). Tasks were grouped to assess participants’ lower extremity function and trunk control, and upper extremity function (Table 1). Scores for upper and lower function were obtained by adding the numeric value received on each of the tasks performed. A higher physical function score indicated less functional ability on the selected tasks. Answer to a single question, one for ambulatory and one for wheelchair use, stood alone to represent general mobility.

**Table 1:** Select ICF mobility activities assessed to calculate mobility function scores relevant to AVG play for each participant.

Category	ICF code	ICF mobility activity	ICF activity description	Test instructions given to participant
**Lower Extremity and Trunk Control**				
	d4101	Squatting	Getting into and out of the seated or crouched posture on one’s haunches with knees closely drawn up or sitting on one’s heels, such as may be necessary in toilets that are at floor level or changing body position from squatting to any other position such as standing up.	“Cross your arms on your chest, and crouch down and touch your buttocks on top of the risers and stand back up. If you can, keep your arms crossed.”
	d4103	Sitting	Getting into and out of a seated position and changing body position from sitting down to any other position, such as standing up or lying down.	Participant able to stand: “Please sit down in this chair and then stand back up.” Participant unable to stand: “Please lie down and then sit up. Now lie back down.”
	d4104	Standing	Getting into and out of a standing position or changing body position from standing to any other position, such as lying down or sitting down.	“Please stand up from the chair and then sit back down.”
	d4105	Bending	Tilting the back downwards or to the side, at the torso, such as in bowing or reaching down for an object.	“Please bend forward and reach for the tape.”
	d4106	Shifting the body’s center of gravity	Adjusting or moving the weight of the body from one position to another while sitting, standing or lying, such as moving from one foot to another while standing.	“Shift your weight over to the left foot (hip if in chair) and come back to the center.” Repeat with the right side.
	d4351	Kicking	Using the legs and feet to propel something away, such as kicking a ball.	“Please kick the ball.”
	d4500	Walking short distances	Walking for less than a kilometer, such as walking around rooms or hallways, within a building or for short distances outside.	“Walk from this piece of tape to the other one at the end of the room and stop when you get there.” Distance is 10 m.
	d4508	Walking other (marching in place)	Not applicable	“While standing bring your knees up to hip level one at a time, like you’re walking up really big steps.”
	d4552	Running	Moving with quick steps so that both feet may be simultaneously off the ground.	“Run from this piece of tape to the other piece of tape at the end of the room.” Distance is 10 m.
	d4553	Jumping	Moving up off the ground by bending and extending the legs, such as jumping on one foot, hopping, skipping, and jumping or diving into water.	“Try to jump over this piece of tape to the other side of the floor.” Colored tape on floor, 1.6 inches wide.
**Upper Extremity Function**				
	d4452	Reaching	Using the hands and arms to extend outwards and touch and grasp something, such as when reaching across a table or desk for a book.	A water bottle is placed on a table. Participant sits just within reaching distance of the table. “Reach for the water bottle and grab it without stepping (rolling) forward.”
	d4401	Grasping	Using one or both hands to seize and hold something, such as when grasping a tool or a door knob.	“Please pick the water bottle up and hold it.”
	d4300	Lifting	Raising up an object in order to move it from a lower to a higher level, such as when lifting a glass from the table.	“Please pick up the water bottle and raise it above your head.”
	d4453	Turning or twisting the hands or arms	Using fingers, hands and arms to rotate, turn or bend an object, such as is required to use tools or utensils.	A water bottle is placed in the participant’s hands. “Please remove the lid from the bottle.”
	d4454	Throwing	Using fingers, hands, and arms to lift something and propel it with some force through the air, such as when tossing a ball.	A tennis ball is placed on the table directly in front of the participant. “Pick the ball up and throw it.”
	d4450	Pulling	Using fingers, hands, and arms to bring an object towards oneself, or to move it from place to place, such as when pulling a door closed.	The participant stands behind a marked spot on the floor. Using green exercise tubing, the tester hands the grip to the participant. Tester takes up the slack of the tubing but does not create tension in the tubing. “Please grab the hand grip and pull it toward you.”
	d4451	Pushing	Using fingers, hands, and arms to move something away from oneself, or to move it from place to place, such as when pushing an animal away.	A rolling chair is placed in front of the participant while they are seated. Tester steps back 4-5 ft and instructs the participant to push the chair away. “Push the chair towards me.”
**Select d4503 OR d465 depending on primary mode of mobility (ambulatory or wheelchair)**				
	d4503	Walking around obstacles	Walking in ways required to avoid moving and immobile objects, people, animals, and vehicles, such as walking around a marketplace or shop, around or through traffic, or other crowded areas.	Three cones evenly spaced in a straight line. “Weave around the cones to the other end and then come back around the cones to the start line.” Distance is 10 m. Participant performs this task walking with assistive device (eg, cane, crutches, walker) if needed.
	d465	Moving around using equipment	Moving the whole body from place to place, on any surface or space, by using specific devices designed to facilitate moving or create other ways of moving around such as with skates, skis, or scuba equipment, or moving down the street in a wheelchair or a walker.	Three cones evenly spaced in a straight line. “Weave around the cones to the other end and then come back around the cones to the start line.” Distance is 10 m. Participant performs this task using their own wheelchair.

In addition to the functional assessment, participants also completed a series of questions from the HealthMeasures resources [38], which were used as an assessment of the individual’s own perspective regarding their functional ability. Questions came from the Patient Reported Outcomes Measurement Information System (PROMIS) and Quality of Life in Neurological Conditions (Neuro-QoL). For adults (18+ years of age), the series comprised questions from PROMIS short-form v1.0 Physical Function 20a and from PROMIS short-form v1.0 Physical Function Samples with Mobility Aid. For youth (10-17 years of age), the questions came from the PROMIS Ped short-form v2.0 Upper Extremity and Neuro-QoL PedScale v1.1‒LE Function (Mobility) scales. For adults, the questions asked how difficult a variety of daily tasks (eg, vacuuming, yard work, walking, bathing) were to complete (5-point scale, “without any difficulty” to “unable to do”, 14 questions), whether their health limited them in their ability to complete certain activities (eg, carry groceries, strenuous sports, walking a mile) (5-point scale, “not at all” to “cannot do”, 6 questions), and could they stand and move with and without support (“yes” or “no”, 1 question; 5-point scale, “without any difficulty” to “unable to do”, 10 questions). For youth, questions asked how difficult a variety of daily tasks (eg, get dressed, open school binder, bend, reach, walk, get on and off low chair) were to complete (5-point scale, “with no trouble” to “not able to do”, 28 questions).

#### Visits 2 and 3

Visits 2 and 3 consisted of exercise testing to assess energy expenditure during AVG play. Upon arrival for these visits, participants were set up with the Cosmed K4B2 portable metabolic system and a Polar heart rate monitor to assess pulmonary gas exchange and indirect calorimetry. Data collection began with a 20-minute rest period to measure resting energy expenditure. For the rest period, participants sat quietly with no speaking or distractions besides light reading of a magazine or viewing their mobile phone. Next, gameplay began with continued Cosmed data collection.

The Nintendo Wii video game console was used for gameplay, with 3 separate video game discs including 1 video game disc for the Wii Balance Board (Wii Fit Plus, Nintendo) and 2 video game discs for the Wii Gaming Mat (Active Life Explorer and Active Life Outdoor Challenge, Bandai Namco Entertainment). Four game sets were created as outlined in Tables 2 and 3. The sets of games played on the balance boards are presented in Table 2, and those played on the gaming mats are presented in Table 3. The games selected for use in this study were chosen in an effort to provide moderate level physical activity during gameplay.

**Table 2:** Description of each AVG played using the OTS and adapted balance boards.

Mini games		Description
**Wii Fit Plus: Set A**		
	Rhythm Kung Fu	The participant follows the Kung Fu movements of avatar characters in time with the rhythm. Movements include left and right punches, two hand punches, and left and right kicks.
	Rhythm Parade	The participant marches in place to the rhythm of the parade while directing the parade with arm movements in coordination with the game.
	Obstacle Course	The participant marches in place to run the character through an obstacle course of swinging balls, moving platforms, and jumps.
	Bird’s-Eye Bull’s-Eye	The participant flaps their arms and moves their body to fly through the course landing on targets spread across the map.
**Wii Fit Plus: Set B**		
	Island Cycling	The participant marches in place on the board to pedal the bike throughout the map while capturing flags that are spread across the island.
	Penguin Slide	The participant catches fish by leaning left and right on the balance board to control the avatar.
	Hula Hoop	The participant rotates their trunk/hips in a circular motion on the board to control the avatar hula hooping.
	Ski Slalom	The participant skis down the slope by leaning left and right to control the avatar skiing the course.

**Table 3:** Description of each AVG played using the OTS and adapted gaming mats.

Mini games		Description
**Active Life Explorer: Set C**		
	Crocodile Stomper	The participant steps on or hits the crocodiles on the game by pressing the corresponding mat button.
	Air Plane Panic	The participant steps on or hits the button prompts as they fly an airplane across a course.
	Kraken Battle	The participant steps on or hits the button prompts as they fight a kraken in attempt to defeat the boss.
	Mummy’s Tomb	The participant runs in place to escape the mummy as the character attempts to escape with the gold.
	Jungle Vine Ruins	The participant runs in place and jumps to navigate the character through the ruins.
**Active Life Outdoor Challenge: Set D**		
	Sprint Challenge	The participant runs in place to sprint and finish the course.
	Jump Rump	The participant jumps in place to control the character jump roping.
	Conveyor Belt Runner	The participant runs in place and jumps over obstacles to navigate the character through the course.
	Log Leaper	The participant jumps in place to control the character jumping over the logs.
	Hurdles	The participant runs in place and jumps to navigate the character through the hurdles.

The controller (Wii balance board or gaming mat), version of the controller (adapted or OTS), and order of the game sets (Board: Game Set A or B; Mat: Game Set C or D) were determined by the participant drawing one of two small pieces of paper out of a cup, which had a number on it (1 or 2). At the start of the second visit, the participant would first draw a number out of the cup to determine which controller (balance board or gaming mat) they would use for that visit. The non-selected controller was played on the subsequent visit. After placing the number back in the cup, the participant would then pull a number to determine which version of the controller they would play first (adapted or OTS). Finally, the participant would draw a number to determine which game set (Board: Set A or B; Mat: Set C or D) they would play first. For the gaming mat, whichever game set was selected first would be played on both versions of the mat (adapted and OTS), and then the second game set would be played on each version. For the balance board, which was difficult to switch between versions because of connectivity and being time consuming, participants played the two set of games on one version (adapted or OTS) and then played on the other version. For each session, gameplay consisted of four 10-minute sets with a rest period of 5 minutes after each game set (Table 4). Visit 3 consisted of the same protocol using the controller that was not played during the previous visit, with numbers drawn for order of play regarding version of the controller and game sets. Data collection for each visit took approximately 75 minutes as follows: baseline energy expenditure‒20 minutes; gameplay‒4 sets x 10 min=40 minutes; rest periods between games‒3 periods x 5 minutes=15 minutes.

**Table 4:** Example of randomized AVG play during balance board session.

Controller	Activity	Time (minutes)
Adapted Balance Board	Gameplay: Set B	10
	Rest	5
Adapted Balance Board	Gameplay: Set A	10
	Rest	5
OTS Balance Board	Gameplay: Set B	10
	Rest	5
OTS Balance Board	Gameplay: Set A	10
	Rest	5

During gameplay, research staff observed and rated ability to use the game controller (mat or board) and quality of gameplay during each visit. Ability to use the game controller assessed the participant’s difficulty/ease of using the controller as required for the game and was rated on a scale of 0-5 (0=Unable, 1=Extreme difficulty, 2=Severe difficulty, 3=Moderate difficulty, 4=Mild difficulty, 5=No difficulty). To assess quality of game play, research staff considered the participants’ degree of general game manipulation and user actions as prompted by the game in comparison to how a gamer without a physical disability would play. Quality of gameplay ranged on a scale from 0-5 (0=Unable, 1=Poor, 2=Fair, 3=Moderate, 4=Good, 5=Excellent). Two research staff worked together for all testing sessions, both recorded scores for quality of gameplay and controller usage, and came to a consensus for the final scores at the end of the session. All sessions were videotaped, so in the event that testers could not come to a consensus, the recording would be available for review.

At the end of each game set, participants reported their rating of perceived exertion on a scale from 0-10, with 0=Not Tired at All and 10=Very, Very Tired. During rest periods, participants completed a feedback survey that included the Physical Activity Enjoyment Scale (PACES) [39]. The PACES includes 16 statements such as “I enjoyed it,” “It was very exciting,” “I felt bored,” and “It was no fun at all.” All items were rated by the participant on a 5-point scale ranging from 1=Strongly Disagree to 5=Strongly Agree. After reverse scoring 7 items, a final score was computed by calculating the average of the 16 items.

## Results

Enrollment started in February 2016 and ended in September 2016. Study results will be reported in late 2017. Outcomes of interest include quality of game play, enjoyment, and energy expenditure. As part of the data analysis, paired comparisons using parametric paired *t* tests will first be conducted. If assumptions of normality are violated then the use of nonparametric tests will be explored. In addition, regression models will be fit that account for covariates such as age and gender. We will evaluate if there is an effect modification by including interaction terms for gender and age. If there is no statistical significance at .05 level for effect modification, an adjustment will be made for these covariates and findings reported based on models that include age and gender as main effects. All statistical testing for prespecified analyses will be conducted at .05 level. Additional post-hoc comparisons will also be conducted and reported. The original *P* value and number of comparisons computed will be reported so that appropriate multiple testing can be performed.

## Discussion

### Principal Considerations

AVGs can provide a fun and engaging activity for improving health and fitness; however, there are technical issues associated with their access and utility for individuals with physical disabilities. The objective of our study is to compare the effect of OTS and adapted game controllers on quality of game play, enjoyment, and energy expenditure during active video gaming in persons with physical disabilities, specifically mobility impairments. The gaming controllers under evaluation include OTS and adapted versions of the Wii Fit balance board and gaming mat.

### Limitations

This is an observational study; therefore, inherent limitations exist and findings will not be generalizable to the broader community based on this study alone. All participants were recruited from the membership of a community physical activity and recreation center for individuals with physical disabilities. All participants were to some degree physically active and varied in their experience with AVGs. Although a familiarization period was provided, some degree of game play learning may have been occurring during data collection. In addition, participants played only a select group of AVGs. Therefore, potential differences in enjoyment and energy expenditure between OTS and adapted controllers may not have been fully captured. Future studies should expand the participant recruitment pool, examine a broader range of AVGs, provide a more extensive familiarization period, and compare AVG play utilizing the adapted controllers to other leisure-time physical activities,

### Conclusions

We hypothesize that the adapted versions of the Wii Fit balance board and gaming mat will produce greater quality of game play, enjoyment, and energy expenditure in persons with mobility impairments compared to OTS versions. Making AVGs accessible to people with disabilities offers an innovative approach to overcoming a number of barriers to participation in physical activity.

## Abbreviations

ACL: Administration for Community Living
AVG: active video game
CP: cerebral palsy
HHS: Department of Health and Human Services
ICF: International Classification of Functioning, Disability and Health
Neuro-QoL: Quality of Life in Neurological Conditions
NIDILRR: National Institute on Disability, Independent Living, and Rehabilitation Research
OTS: off-the-shelf
PACES: Physical Activity Enjoyment Scale
PROMIS: Patient Reported Outcomes Measurement Information System
SCI: spinal cord injury
